# The Impact of Artificial Intelligence on Women’s Healthcare: A Systematic Review

**DOI:** 10.5339/qmj.2026.36

**Published:** 2026-06-23

**Authors:** Saheed Akinola Shittu, Hanen Mrabet, Sufia Athar, Mohamed Ezzeldin Gaber, Tamara Alshdafat, Salwa Alrawaili, Parwaneh Shibani, Lolwa Alansari, Moayyad Younis

**Affiliations:** 1 Hamad Medical Corporation, Doha, Qatar

**Keywords:** Artificial intelligence, machine learning, women’s health, obstetrics and gynecology, clinical application, women’s healthcare

## Abstract

**Background:**

Artificial intelligence (AI) is rapidly transforming healthcare delivery with substantial implications for women’s health. This systematic review synthesizes current evidence on AI applications in women’s healthcare, evaluates their contributions and limitations, and identifies key challenges for clinical implementation.

**Methods:**

A systematic analysis of peer-reviewed literature was conducted through database searches, including PubMed, Scopus, Web of Science and the Cochrane Library, focusing on AI applications in the Obstetrics and Gynecology domains.

**Results:**

The analysis reveals extensive AI development across obstetrics and gynecology subfields, particularly in obstetric imaging, fetal monitoring, gynecologic oncology, and predictive models for delivery outcomes. Both machine learning (59% of studies) and knowledge-based systems (38% of studies) are represented. Most publications (82%) represent preliminary work such as proof-of-concept algorithms or methods, with clinical validation remaining largely unreported. Key implementation challenges include limited external validation, ethical concerns, and the need for specialized clinician competencies.

**Conclusion:**

AI demonstrates significant potential to enhance diagnostic precision, personalized treatment, and support clinical decision-making in women’s health. However, most applications remain investigational with substantial barriers to clinical translation. Future work should prioritize robust validation, standardized reporting, and interdisciplinary collaboration to realize AI’s potential in women’s healthcare.

## 1. INTRODUCTION

John McCarthy, an American computer scientist regarded as one of the founders of artificial intelligence (AI), defined AI as the science and engineering of making intelligent machines.^1^ AI has also been defined as the capability of computational systems to perform tasks typically associated with human intelligence, such as learning, reasoning, problem-solving, perception, and decision-making. It is a field of research in computer science that develops and studies methods and software that enable machines to perceive their environment and use learning and intelligence to take actions that maximize their chances of achieving defined goals.^2^

The integration of AI into healthcare represents a paradigm shift in medical practice, offering unprecedented capabilities in data analysis, pattern recognition, and clinical decision support.^3^

**Figure 1.**
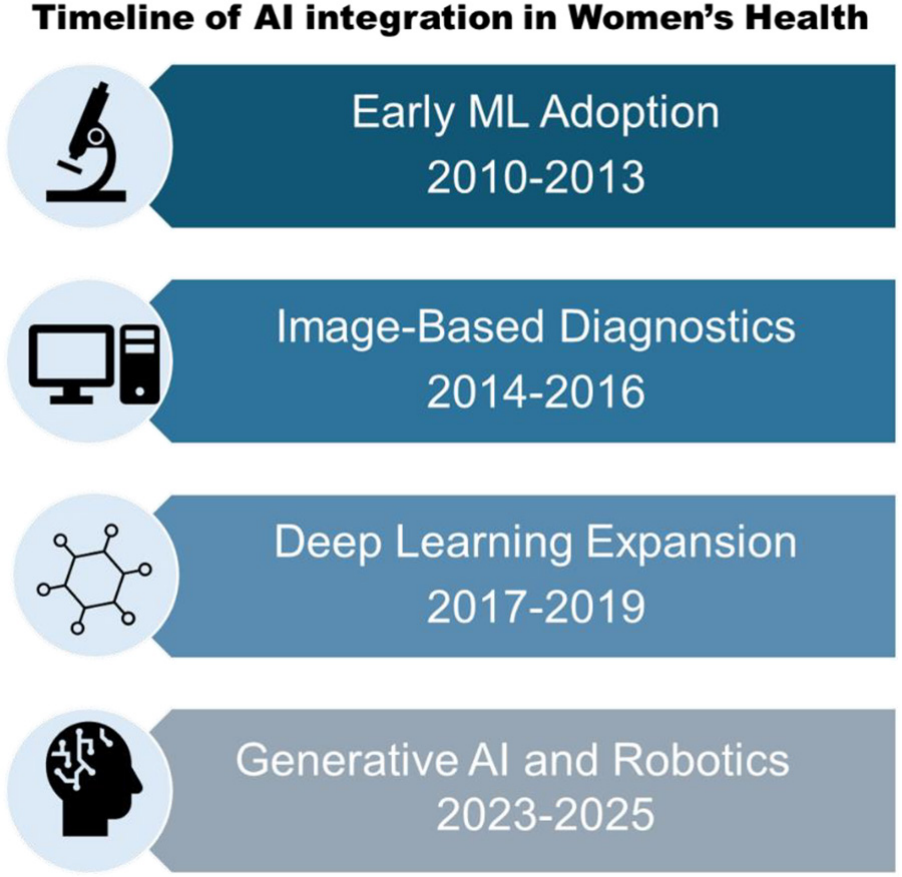
AI integration in women’s health.

In obstetrics, gynecology, and reproductive medicine, AI applications have expanded dramatically over the past two decades, spanning diverse domains from prenatal imaging to gynecological oncology and reproductive medicine. The growing publication trend reflects increasing interest in AI’s potential to address complex challenges in women’s health, with applications ranging from the automated classification of fetal brain ultrasound images as normal or abnormal to predict models for obstetric complications and diagnostic support for gynecologic cancers.^4,5^ The timeline of AI’s integration into women’s health is shown in Figure 1.

Machine learning (ML) models are generally classified into three broad categories: supervised, unsupervised, and reinforcement learning models, each suited to different tasks in clinical practice. In supervised learning, models are trained on labelled data. Based on this training, the model can make predictions for new, unseen data. Common supervised learning models include logistic regression, decision trees, and neural networks.^6^ These models have been widely applied in obstetrics, gynecology, and reproductive medicine, ranging from prognosis, such as pregnancy complication prediction and fetal abnormality detection, to the success rate evaluation of assisted reproductive techniques.^8–10^

Unsupervised learning models, on the other hand, do not require labelled data; instead, the model identifies patterns and structures within the dataset on its own. Many unsupervised models, such as clustering algorithms (k-means and hierarchical clustering) and dimensionality reduction techniques (principal component analysis, PCA), can segment patient groups based on risk profiles or response patterns. Unsupervised models excel at discovering new knowledge and segmenting patient groups, which can lead to more personalized and effective treatment strategies.^11^

**Figure 2.**
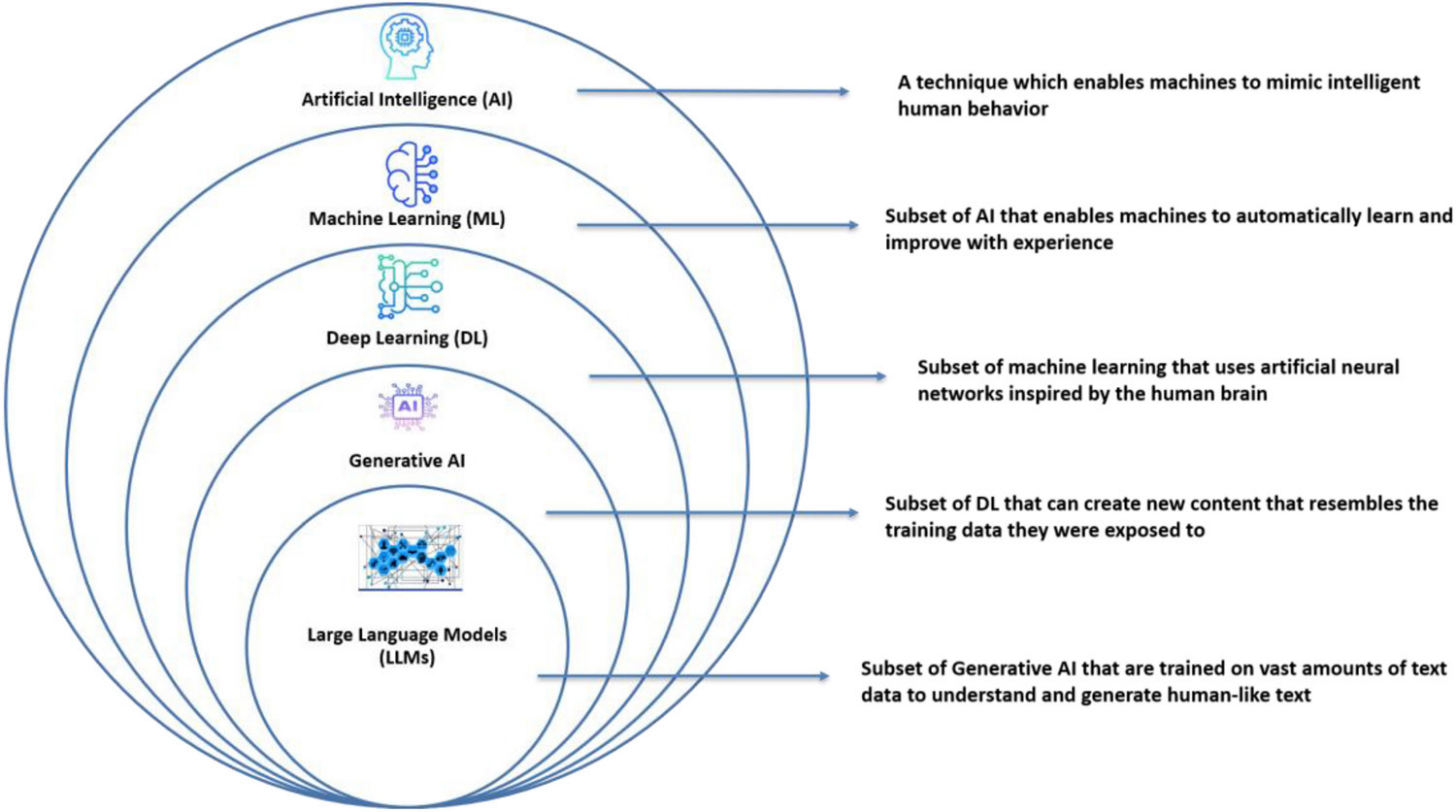
AI model concepts.

**Figure 3.**
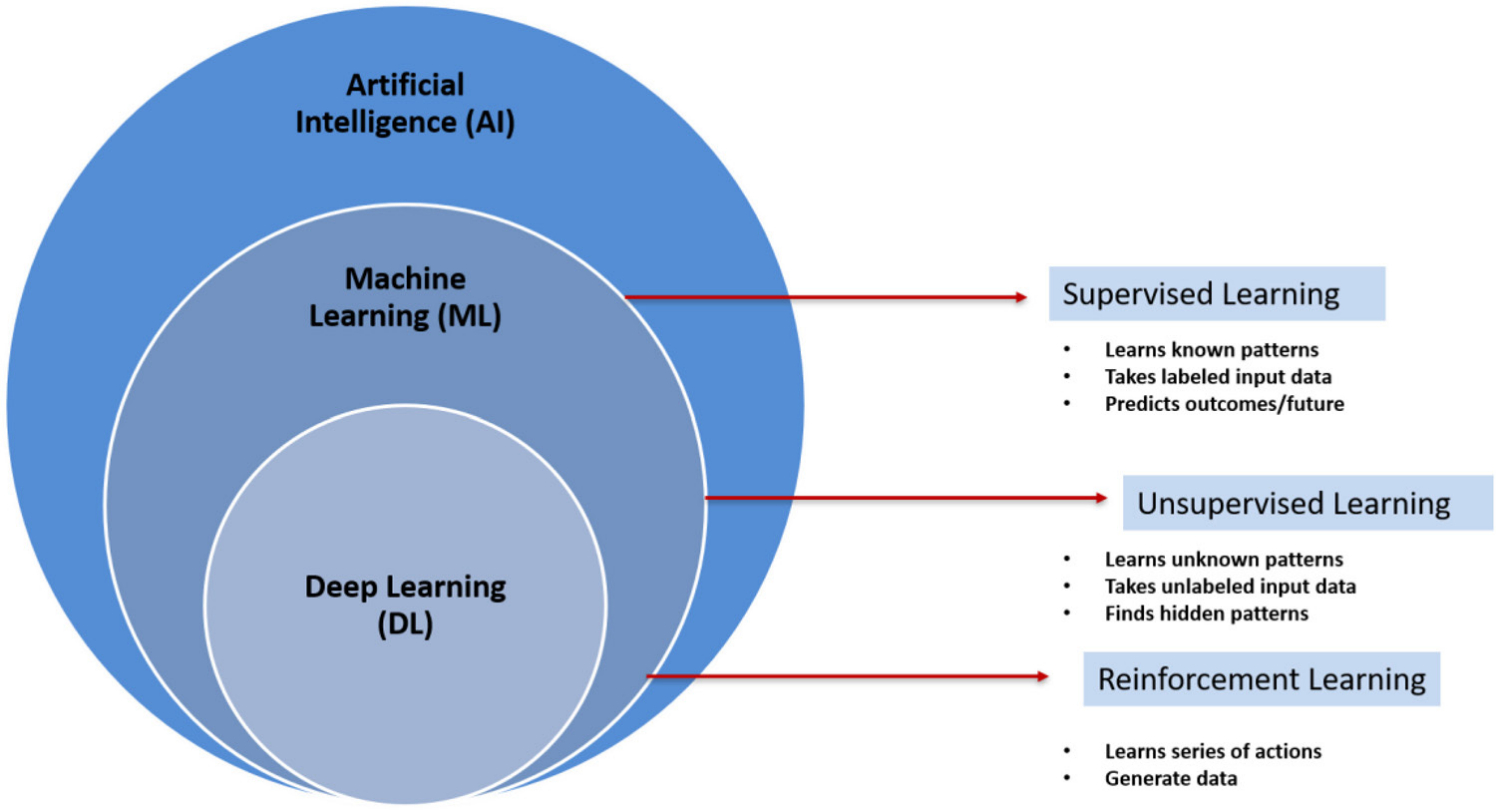
Types of machine learning algorithms.

Reinforcement learning models are inspired by the idea of learning from experience and mistakes. Reinforcement learning differs from supervised and unsupervised approaches in that it involves an agent that learns through trial and error by interacting with its environment. The agent receives feedback in the form of rewards or penalties based on its actions, allowing it to progressively optimize decision-making. This learning process mirrors experiential human learning and is particularly suited for complex, dynamic clinical environments.^12^ AI concepts and types of ML algorithms are shown in Figures 2 and 3.

This systematic review aims to critically evaluate the current landscape of AI applications in women’s healthcare, focusing on established use cases, methodological approaches, validation experiences, and implementation challenges. It provides a comprehensive assessment of AI’s influence across the breath of women’s healthcare, with particular attention to the translational gap between algorithm development and clinical integration.

## 2. METHODOLOGY

### 2.1. Search strategy and selection criteria

This Systematic Review was conducted following the preferred reporting items for systematic reviews and meta-analysis (PRISMA) guidelines. The protocol for this systematic review was pre-registered with the International Prospective Register of Systematic Reviews (PROSPERO; registration number [CRD42024527916]). We performed a comprehensive literature search across multiple electronic databases, including PubMed, Scopus, Web of Science, and the Cochrane Library. The search strategy incorporated structured queries combining terms related to AI (“Artificial Intelligence” OR “machine learning” OR “deep learning” OR “neural networks”) with obstetrics and gynecology terminology (“obstetrics” OR “gynecology” OR “pregnancy” OR “reproductive medicine” OR “gynecologic oncology”). The search encompassed publications from January 2010 through December 2023 to capture the evolving landscape of AI in obstetrics and gynecology. The detailed search strategy is presented in Box 1.

**Box 1.**

Search Strategy: (“Artificial Intelligence” OR “Machine Learning” OR “Deep Learning” OR “Neural Networks”) AND (“Obstetrics” OR “Gynecology” OR “Fertility” OR “Reproductive Medicine”) AND (“Diagnosis” OR “Risk Prediction” OR “Clinical Decision Support” OR “Treatment”).

### 2.2. Study selection and data extraction

Studies were included if they reported on the development, validation, or implementation of AI technologies in obstetric or gynecologic care. The inclusion criteria encompassed: (1) primary research studies; (2) focus on obstetrics and gynecology populations or clinical scenarios; (3) a description of AI/ML methodology; and (4) reporting of performance metrics or implementation outcomes. The exclusion criteria included: (1) reviews, articles, editorials, and commentaries; (2) studies not specific to obstetrics and gynecology; (3) non-English publications; and (4) studies with insufficient methodological details.

**Figure 4.**
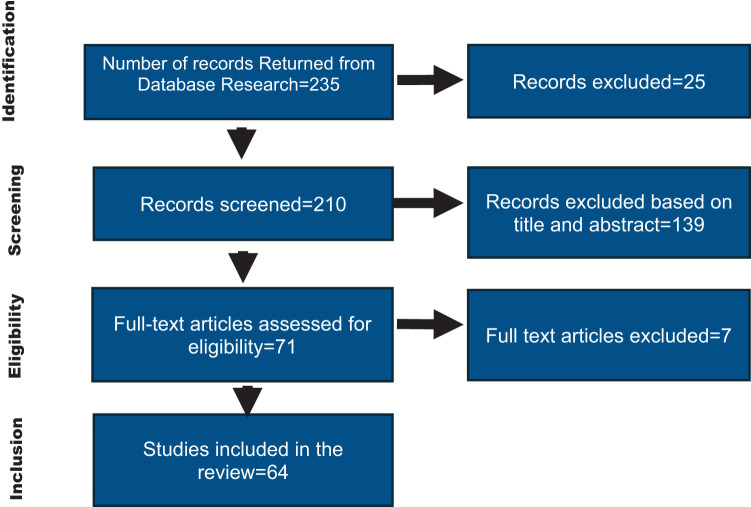
PRISMA flowchart.

The study selection process involved title and abstract screening, followed by full -text review of potentially eligible articles. Data extraction captured information on study characteristics (author, year, and design), AI methodology (type of algorithm and data resources), clinical application, performance metrics, and validation approach. The quality assessment of the included studies was evaluated using appropriate tools, such as PROBAST for prediction model studies and CONSORT guidelines for randomized trials, where applicable. Figure 4 shows the PRISMA flowchart.

## 3. AI APPLICATIONS ACROSS OBSTETRICS AND GYNECOLOGY SPECIALTIES

### 3.1. Overview of AI applications

AI applications in women’s health span the entire spectrum of women’s healthcare, from prenatal care to gynecologic surgery and oncology. A systematic review of 64 studies published in obstetrics and gynecology journals between 2010 and 2023 reveal that obstetric applications constituted the largest proportion (41%), followed by assisted reproductive medicine (33%), fetal medicine (21%), gynecology (3%), and early pregnancy (2%). Both ML methods (59% of studies) and knowledge-based systems (38% of studies) were well represented, indicating diverse methodological approaches to AI implementation in women’s health. Table 1 shows the distribution of AI applications in obstetrics and gynecology specialties.

### 3.2. AI in obstetric imaging and diagnostics

Prenatal ultrasound has emerged as a major application area for AI, particularly deep learning algorithms.^4,13,14^ Convolutional neural networks (CNNs) have demonstrated remarkable capabilities in automating standard fetal biometric measurement from ultrasound images, performing at levels comparable to expert sonographers while potentially reducing scan time by up to one-third. These systems can automatically compute key measurements such as biparietal diameter, head circumference, abdominal circumference, and femur length without manual input, thereby standardizing assessment and reducing operator dependency.^14–16^

Beyond routine biometry, AI shows significant promise in prenatal anomaly detection.^17^ For central nervous system (CNS) abnormalities, a convolutional U-Net combined with a Visual Geometry Group network demonstrated a 97.5% reduction in false negatives for brain anomalies, achieving approximately 96–97% correct classification of normal vs. abnormal brain anatomy.^18,19^ Similarly, the prenatal ultrasound diagnosis AI conduct system demonstrated high diagnostic accuracy for fetal CNS abnormalities in both image datasets and real-time scans, matching expert performance while improving time efficiency.^18,20–22^ These advances suggest AI’s potential as a decision-support tool in busy clinical settings where specialist expertise may be limited.^13,14,21–23^

### 3.3. AI in fetal monitoring and risk prediction

Electronic fetal monitoring through cardiotocography (CTG) generates complex data streams that benefit from AI-driven analysis. Traditional CTG interpretation suffers from significant inter-observer variability, which AI approaches aim to reduce. Deep learning models trained on nearly 19,400 CTG recordings matched with cord blood pH and APGAR outcomes have shown promise in predicting fetal hypoxia, potentially providing more consistent interpretation and earlier alerts for fetal distress.^6,7^ Other deep CNN models for CTG analysis have achieved performance on par with expert clinicians (area under the curve [AUC]: 0.68–0.70), highlighting the feasibility of algorithmic prediction of fetal compromise.^23–25^

AI has also been extensively applied to obstetric risk prediction.^22^ Multiple ML models have been developed to predict conditions such as preeclampsia, with a recent review reporting AUC values ranging from 0.86 to 0.96 across studies.^26–28^ These models incorporate diverse data sources, including maternal history, laboratory results, and ultrasound markers, to generate individualized risk assessments.^29,30^ Similar approaches have been applied to predict preterm birth^22,31–33^ and postpartum hemorrhage (PPH),^34^ often outperforming traditional scoring systems. Notably, ML models trained on electronic health records from 29,000 mothers have successfully identified women at high risk of postpartum depression, with flagged individuals being nearly three times more likely to develop the condition compared to the average population.^35,36,37^

### 3.4. AI in gynecologic imaging and cancer screening

In gynecologic oncology, AI demonstrates particular strength in diagnostic imaging.^38–40^ A large multicentre study with 17,119 images from 3,652 patients across eight countries developed transformer-based neural networks to distinguish benign from malignant ovarian lesions on ultrasound. The AI model generalized well across centres and significantly outperformed both expert and novice clinicians in sensitivity, specificity, and overall accuracy. In clinical triage simulations, AI reduced referral to specialists by 63% while maintaining diagnostic quality, suggesting its potential to alleviate shortages of expert sonographers.^41–45^

Cervical cancer screening has also benefited from advances in AI. One study developed an AI system for cervical cytology screening trained on more than 16,000 cases, achieving an AUC of 0.947 for detecting high-grade lesions.^46–48^). When used to assist cytopathologists, AI significantly improved sensitivity by 13.3%, demonstrating its value as a digital second reader in cancer screening.

In breast care, AI-supported mammography has demonstrated a 17.6% higher cancer detection rate in real-world trials compared to standard radiologist readings. It acts as a “second set of eyes”, identifying subtle abnormalities and reducing false positives by up to 5.7%.^49^ AI triaging can safely remove up to 60% of normal scans from a radiologist’s workload, allowing specialists to focus on high-risk cases. Advanced models can now predict long-term cancer risk by analysing subtle imaging biomarkers years before clinical symptoms appear.^50^ Beyond cancer, AI tools are being developed to segment uterine fibroids or the endometrial lining, on ultrasound and MRI, quantify volumes, and predict treatment responses, although these applications remain largely investigational.^51,52^

### 3.5. AI in menopause and midlife women’s health

AI is bridging the historical “gender health gap” in menopause. Platforms like Hormona use AI-driven hormone tracking and symptom analysis to provide tailored interventions and support for perimenopausal women. ML models have been applied to large clinical datasets to identify the factors driving vasomotor symptoms, such as hot flushes, enabling more nuanced prediction and understanding of symptom burden beyond traditional approaches. AI models can also identify women at higher risk for menopause-related complications, such as osteoporosis and cardiovascular disease.^53^

### 3.6. AI in delivery prediction and surgical planning

Delivery route prediction represents an emerging application with significant implications for resource allocation and patient counselling.^5^ A systematic review of 17 studies on AI models for predicting mode of delivery found that ensemble methods and advanced ML techniques generally outperformed traditional logistic regression models.^9,14,20,32^ These models incorporated variables such as maternal age, parity, BMI, previous caesarean, sonographic findings, and cervical examination data. Real-time intrapartum data significantly enhanced model accuracy in several studies, although model transparency and external validation were identified as critical considerations for clinical translation.^13,21–23^

In gynecologic surgery, AI integration is advancing, particularly in robotic-assisted procedures. Modern robotic platforms already incorporate advanced software control, with the next step being the embedding of ML capabilities.^41,43–45^ AI algorithms can process endoscopic video feeds to identify anatomy or pathology, providing real-time analytics and guidance to surgeons, thereby enhancing surgical precision and reducing complications.^39,43^

In parallel, AI has been increasingly explored in reproductive medicine and assisted reproduction laboratories. ML models have been applied to embryo and oocyte classification, implantation prediction, and clinical pregnancy forecasting, often outperforming conventional statistical approaches.^54–56^ Recent reviews and comparative studies suggest that AI-based embryo selection systems can match or exceed embryologist performance, supporting their role in optimizing treatment outcomes and standardizing laboratory decision-making.^57–66^ Additional applications include the prediction of infertility risk and the optimization of controlled ovarian stimulation protocols using supervised learning techniques.^65^ Table 2 shows the performance metrics of selected AI applications in Obstetrics and Gynecology.

## 4. CLINICAL VALIDATION AND EVIDENCE QUALITY

### 4.1. Current validation status

Despite the proliferation of AI applications in women’s health, the validation landscape remains concerning. A systematic review of 64 studies found that validation was typically performed on a single dataset (86% of studies), with no external validation reported in any of the included publications. This lack of robust validation represents a significant barrier to clinical implementation, as algorithm performance may degrade when applied to populations or settings different from those used in the development. The same review noted that most publications (82%) remain outside the scope of usual Obstetrics and Gynecology journal, potentially limiting the exposure to clinical audiences.

The evidence quality for AI applications in women’s health varies substantially across domains. In a systematic review of AI’s impact on educational outcomes in health professions education, researchers found poor methodological quality among included studies, with heterogenous designs, uncontrolled confounding variables, and insufficient information on training datasets. Similar limitations likely affect many clinical AI studies, highlighting the need for more rigorous evaluation frameworks specifically tailored to AI technologies in healthcare settings.^49–51^

### 4.2. Implementation challenges

The integration of AI tools into clinical workflow faces several significant barriers.

: A qualitative synthesis of studies of healthcare professionals’ experiences with AI tools identified key implementation barriers, including: (1) limited understanding of AI applications; (2) lack of trust and confidence; (3) uncertainty regarding clinical value; (4) data availability constraints; (5) time and workload pressures; (6) governance and liability concerns; and (7) the need for multidisciplinary collaboration.^67–69^ Many healthcare professionals reported concerns about not understanding AI outputs or the rationale behind them, leading to reduced confidence in the accuracy of AI applications and their recommendations.

: Issues of data governance, responsibility for errors, and security have been widely noted. The rapid evolution of AI technologies has outpaced existing regulatory frameworks, leading to ongoing uncertainty regarding approval processes for AI-based algorithms in medicine. In addition, concerns about algorithmic bias, data inclusiveness, and equity in access to AI -enhanced care require careful attention as these technologies advance toward clinical implementation.

## 5. DISCUSSION

### 5.1. Summary of evidence

This systematic review demonstrates the substantial potential of AI to transform multiple aspects of obstetrics and gynecology care. Current applications span the entire spectrum of women’s health, from prenatal diagnosis to gynecologic oncology, with particularly strong performance in image-based tasks such as fetal anomaly detection and ovarian mass classification. The evidence suggests that AI can enhance accuracy, reduce human error, standardize assessment, and potentially improve workflow efficiency in Obstetrics and Gynecology practice.

However, our analysis reveals a significant translational gap between algorithm development and clinical implementation. Most publications describe proof-of-concept algorithms or methods rather than clinically validated tools ready for routine use. The almost universal absence of external validation raises concerns about generalizability, while limited information on integration with clinical workflow hinders practical implementations. Furthermore, the concentration of AI development in specific domains such as obstetrics and assisted reproduction leaves other areas of gynecology relatively underexplored.

### 5.2. Implementation consideration

Successful integration of AI into Obstetrics and Gynecology practice will require addressing several key challenges. First, clinician acceptance depends on improving the understanding of AI systems. Healthcare professionals need appropriate education about AI fundamentals, including how algorithms are developed and validated, their limitations, and appropriate interpretation of output. Developing these competencies should be incorporated into both the undergraduate medical education and continuing professional development programs.

Secondly, regulatory and ethical frameworks must evolve to ensure patient safety while promoting innovation. Clear guidelines are needed for data governance, algorithm validation, accountability structures, and post-market surveillance of AI tools. Particular attention should be paid to mitigating algorithmic biases that could exacerbate health disparities, ensuring that AI technologies benefit diverse patient populations.

Thirdly, workflow integration requires careful design to augment rather than disrupt clinical practice. AI tools should be seamlessly embedded into existing clinical systems with intuitive interfaces and clear presentation of actionable information. Implementation should be accompanied by robust change management strategies that engage all stakeholders and address concerns about professional autonomy, workflow modification, and the potential deskilling of clinical judgment.

### 5.3. Future directions

Based on our analysis, we recommend several priority areas for future work in AI for women’s health.

1. : Develop and adopt standardized reporting guidelines for AI studies in women’s health, including mandatory external validation on independent datasets and prospective evaluation of clinical utility.
2. : Increase research on implementation strategies, including workflow integration, user interface design, and change management approaches specific to AI technologies in women’s health settings.
3. : Incorporate AI literacy into women’s health training programs, focusing on critical appraisal of AI technologies, ethical considerations, and effective human–AI collaboration.
4. : Prioritize development of diverse, representative datasets to minimize algorithmic bias and to ensure that AI technologies benefit all patient populations.
5. : Foster closer collaboration among clinicians, computer scientists, engineers, and implementation specialists to ensure that AI solutions address genuine clinical needs with appropriate methodological rigor.

## 6. CONCLUSION

Artificial intelligence holds transformative potential for obstetric and gynecologic care, with demonstrated capabilities in enhancing diagnostic precision, personalizing treatment approaches, and supporting clinical decision-making. Current applications span the breadth of women’s health, from prenatal imaging to gynecologic oncology, although development has been uneven across subspecialties. Despite promising performance metrics in controlled settings, most AI tools remain investigational, with limited validation and implementation evidence.

Bridging the gap between algorithm development and clinical integration will require addressing substantial challenges related to validation, regulation, workflow integration, and clinician acceptance. Future work should prioritize robust validation frameworks, interdisciplinary collaboration, and thoughtful implementation strategies to realize AI’s potential to improve women’s healthcare outcomes. With appropriate attention to these considerations, AI technologies may ultimately transform obstetric and gynecologic practice, enhancing both the art and science of women’s healthcare.

## LIST OF ABBREVIATIONS

**Table:** 

AI	Artificial Intelligence
AUC	Area Under the Curve
CONSORT	CONsolidated Standard of Reporting
CNN	Convolutional Neural Network
CNS	Central Nervous System
CTG	Cardiotocography
DL	Deep Learning
ML	Machine Learning
PCA	Principal Component Analysis
PRISMA	Preferred Reporting Items for Systematic Reviews and Meta-Analyses
PROBAST	Prediction model Risk of Bias Assessment Tool

## DATA AVAILABILITY

No datasets were generated or analyzed during the current study.

## ETHICAL APPROVAL

Not applicable.

## AUTHORS’ CONTRIBUTION

SS, HM, SA, and MG: Conceptualization; SS, HM, and SA: Data curation, Methodology and Project administration; SS, HM, LA, and MY: Supervision; SS and HM: Visualization; SS, HM, SA, MG, TA, SA, PS, LA, MY: Formal analysis, Investigation, Validation, Writing – original draft, and Writing – review & editing.

## FUNDING

Open Access funding provided by the Hamad Medical Corporation. The authors received no financial support for the research, authorship, or publication of this article.

## ACKNOWLEDGEMENTS

We thank Hamad Medical Corporation for funding the open access publication of this article.

## DECLARATION OF AI USE

The authors acknowledge the use of artificial intelligence-assisted tools in refining the language, improving the academic tone, and enhancing the readability of this manuscript. All scientific content, data interpretation, and conclusions were conceived, written, and verified by the authors.

## CONSENT FOR PUBLICATION

Not applicable.

## CONFLICT OF INTEREST

The author(s) declare that there is no conflict of interest.

## Figures and Tables

**Table 1.** Distribution of AI applications in obstetrics and gynecology specialties.

Specialty Domains	Percentage of Studies	Primary Applications	Common Data Sources
General Obstetrics	41%	Risk prediction, delivery outcome forecasting	Clinical data, electronic health records
Assisted Reproductive Medicine	33%	IVF success prediction, embryo selection	Omics data, imaging, clinical parameters
Fetal Medicine	21%	Anomaly detection, biometry, fetal monitoring	Ultrasound, MRI, cardiotocography
Gynecology	3%	Surgical planning, benign condition diagnosis	Clinical notes, imaging, and surgical videos
Early Pregnancy	2%	Pregnancy outcome prediction	Ultrasound, biomarker data

**Table 2.** Performance metrics of selected AI applications in Obstetrics and Gynecology.

Application Area	Reported Performance	Comparison to Standard Care
Fetal CNS anomaly detection	96–97% correct classification; 97.5% reduction in false-negative findings	Matches expert performance with improved time efficiency
Ovarian mass classification	Significantly higher sensitivity, specificity, and overall accuracy compared with clinicians	63% reduction in specialist referrals while maintaining diagnostic quality
Cervical cytology screening	AUC up to 0.947 for detection of high-grade lesions; 13.3% increase in sensitivity when assisting cytopathologists	Superior performance compared with manual screening alone
Pre-eclampsia prediction	AUC ranging from 0.86 to 0.97 across studies	Outperforms traditional clinical risk scores; enables targeted preventive and early intervention
Postpartum depression prediction	Nearly threefold higher risk identified in AI-flagged populations	Supports earlier identification of at-risk populations
Breast cancer screening (mammography)	17.6% higher cancer detection rate; reduction in false-positive recalls by up to 5.7%; safe exclusion of up to 60% of normal scans from radiologist review	Functions as a second reader, improves diagnostic accuracy, and reduces radiologist workload
Breast cancer risk prediction	Accurate long-term risk prediction using subtle imaging biomarkers years before clinical presentation	Enables earlier risk stratification beyond traditional questionnaires
Benign gynecologic imaging (fibroids, endometrium)	Automated segmentation and volumetric quantification using ultrasound and MRI	Promising investigational tools; clinical utility and treatment-response prediction under evaluation
Menopause and midlife women’s health	Machine learning models identify factors associated with vasomotor symptoms and stratify risk for menopause-related complications	Supports personalized symptom management and proactive midlife health interventions
